# Evaluation of Intrauterine Structural Pathology by Three-Dimensional Sonohysterography Using An Extended Imaging Method

**Published:** 2013-03-06

**Authors:** Fatemeh Zafarani, Firoozeh Ahmadi

**Affiliations:** Department of Reproductive Imaging at Reproductive Biomedicine Research Center, Royan Institute for Reproductive Biomedicine, ACECR, Tehran, Iran

**Keywords:** Intrauterine, Pathology, Three-Dimensional, Sonohysterography

## Abstract

Structural intrauterine abnormalities are an important cause of infertility, recurrent pregnancy loss and bleeding or pain associated with a poor reproductive outcome. Various diagnostic methods have been applied to detect these lesions such as hysterosalpingography, hysteroscopy and sonohysterography. More recently, three-dimensional extended imaging (3DXI) provides the ability to obtain sequential sections of acquired volume scans in A, B and C planes. Here, we briefly discuss the technique of saline infusion sonography, followed by a review of sonohysterographic characteristics of intracavitary pathologies with more focus on some definitions and measurements.

## Introduction

In addition to visualizing normal tissue, imaging studies of the uterine cavity can also show the presence of reactive, inflammatory, benign and malignant neoplastic tissue. Structural abnormalities of the uterus are an important cause of infertility, recurrent pregnancy loss and poor reproductive outcomes (1, 2). Intracavitary lesions are additionally the cause of other problems such as abnormal bleeding or pain. An intracavitary abnormality can be detected by several methods such as hysterosalpingography, ultrasonography, hysteroscopy, magnetic resonance imaging (MRI) and sonohysterography. Currently, diagnostic hysteroscopy is considered the preoperative investigation of choice (3). Despite providing direct visualization of the uterine cavity via hysteroscopy, limitations may favor the use of other precise diagnostic tools such as sonohysterography (4, 5). Previous studies have shown that two-dimensional sonohysterography is less invasive, less expensive, more comfortable, less complicated and less time consuming than hysteroscopy for detecting intracavitary abnormalities (6-8). In the 1990s, three-dimensional ultrasound that has the ability to visualize uterine morphology in the coronary plane and provide an accurate diagnosis or exclusion of intracavitary lesions was introduced.

Recently, three-dimensional extended imaging (3DXI), which is a powerful computed processing technique similar to CT and MRI has provided the ability to obtain sequential sections of acquired volume scans in A, B and C planes. Image thickness in the series can be manipulated by fractions of a millimeter to a few millimeters and the interval of slices varies in the accordance with the area of interest and is dependent upon the volume size. The pictures can be obtained in the form of a contiguous series of thin slices [multi-slice (MS) view] or strong multi-resolution images. Here, we briefly discuss the technique of saline infusion sonography, followed by a review of the 3DXI sonohysterographic characteristics of intrauterine lesions such as polyps, leiomyomas, hyperplasia, and intra-cavitary adhesions.

### Technique of saline infusion sonography

#### Timing and patient preparation

The examination is typically scheduled in the early follicular phase of the menstrual cycle, immediately after cessation of menstrual flow and before day ten. The endometrium is relatively thin during the early proliferative phase of the cycle, which facilitates imaging interpretation. In the late luteal phase of the cycle, thickened endometrium or focal irregularities in the endometrial outline may be mistaken for endometrial hyperplasia or small polyps.

Prophylactic antibiotics are not administered unless there is a history of chronic pelvic inflammation. The majority of patients can tolerate the procedure and anesthesia or analgesia is not usually required for catheter insertion. In cases that experience some cramping, a nonsteroidal anti-inflammatory medication such as ibuprofen (400 mg) is prescribed 30 minutes prior to the examination.

#### Procedure

A preliminary transvaginal sonography is recommended to investigate the uterus and adenexes for any abnormal findings. The procedure must be performed under strict conditions since saline, after it passes the genital tract, may introduce infection into the peritoneal cavity.

A suitable sized speculum is inserted into the vagina and the cervix is cleaned with antiseptic solution. A Foley 6-Fr pediatric bladder drainage catheter (Supa Co., Tehran, Iran) is introduced into the cervical canal and a balloon at the catheter tip is placed in the lower uterine segment or cervical canal and inflated with 1 mL of sterile saline solution. The speculum is removed and a covered vaginal probe is inserted. Under sonographic guidance, sterile normal saline solution (5-10 mL) is slowly introduced into the cavity. Three-dimensional ultrasound volume scanning is then performed using a high-resolution three-dimensional ultrasound machine (5-8 MH probe, Accuvix XQ, Medison, Korea). When optimal distention of the endometrial cavity is achieved, a three-dimensional volume sweep of the sagittal and transverse planes of the uterus are performed. Scanned volumes are evaluated in multi-planar three-dimension and MS view mode with a slice interval of 0.5-0.6 mm.

Since the distended balloon may obscure pathology it is deflated immediately before the end of the procedure, after which the catheter is slowly withdrawn while adding more fluid to ensure adequate visualization of the lower uterine segment and cervical canal. Offline analyses of uterine morphology and the endometrial cavity are performed in a reconstructed coronal plane. Uterine structure, particularly the contour of the uterine fundus and any focal or diffuse endometrial or subendometrial abnormalities are analyzed in each patient. Congenital uterine anomalies, if present, are classified according to the American Fertility Society Classification.

### Sonohysterographic findings

#### Endometrial polyp

**Fig 1:**
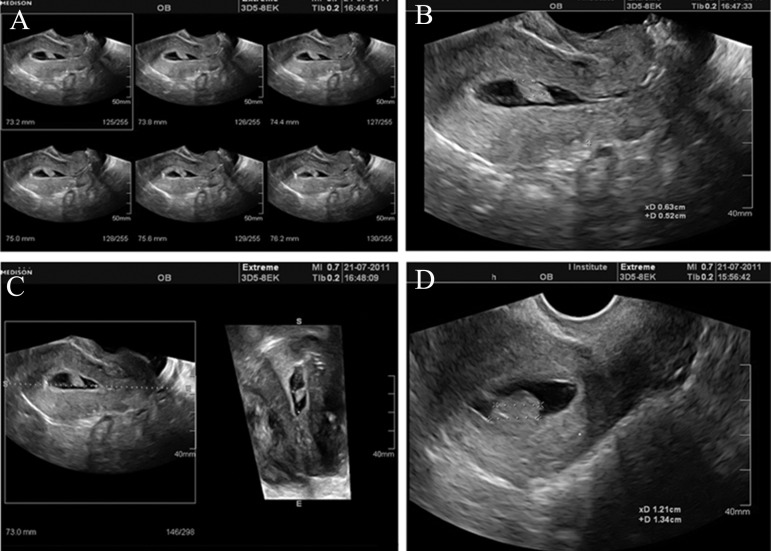
3D-MS- View of endometrial outline in the transverse plane shows a localized lesion (2×3, slice interval 0.6 mm). A. Optimal image of the lesion is obtained by scrolling the parallel planes of volume. B. a/b ratio <1 indicating " pedunculated" lesion. C. Coronal reconstructed view confirmed the pedunculated polyp. D. a/b ratio≥1 indicating " sessile" lesion. " a" is the maximum diameter of the base of the lesion at the level of the endometrium and " b" is the maximum transverse diameter of the lesion.

Endometrial polyps that appear as a solitary or multiple, diffuse or focal, sessile or pedunculated thickening of the endometrium are the most common anomalies visualized on sonohysterography. An endometrial polyp may occur either alone, in the setting of endometrial hyperplasia or less commonly, carcinoma (9). By using sonohysterography, intracavitary polyps are usually seen as isoechoic, relative to the endometrium. Some are nonuniform with small internal cysts. Color/power Doppler findings show the presence of a single feeding vessel which distinguishes polyp from the myoma, while myomas have several vessels that arise from the inner myometrium. Sonohysterography enables us to evaluate the number and size of polyps in greater detail. In 3DXI sonohysterography, multiple parallel planes with adjustable distances (2-5 mm) provide the ability to visualize the polyp at its widest diameter. Offline analysis of the endometrial cavity in a reconstructed transverse and coronal plane allows the technician to measure the diameter of the base at the level of the endometrium or "a" and the maximal transverse diameter of the lesion or "b". The ratio of a/b determine the type of the polyp, which is defined as "pedunculated" if the ratio is <1 and "sessile" if it is 1 or more (10) (Fig 1 A-D).

### Submucosal leiomyoma

Leiomyomas are the most common benign pelvic tumors of the female genital tract, occurring in 20-25% of reproductive-age women (11, 12). Myomas consist of smooth muscle and varying amounts of soft tissue. The location of a submucosal leiomyoma can be easily detected in sonohysterography by its broad-based appearance, echogenecity and the proportion of the myoma that protrudes into the uterine cavity (10, 13, 14). Myomas are hypo-to isoechoic relative to the myometrium, whereas polyps are isoechoic relative to the endometrium. The echogenecity of submucosal and intracavitary myomas may be uniform or non-uniform. In color/power Doppler findings circular flow is present.

**Fig 2:**
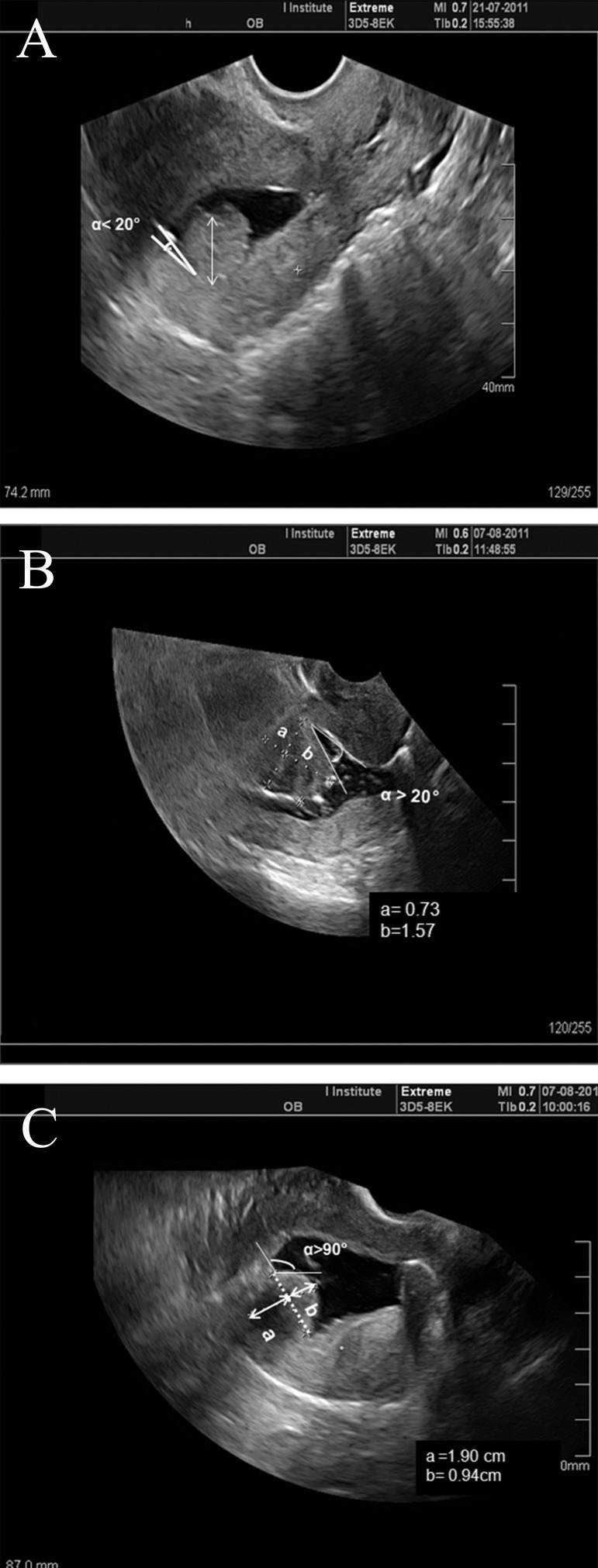
3D-MS-SHG shows a submucousal myoma in longitudinal section of the uterus. A. Completely intracavitary fibroid, pedunculated, without intramural extension, angle ≤20˚. B. Protrusion index (>50% and angle≤90˚) suggestive of Type I submucous myoma. C. Protrusion index (<50% and angle>90˚) suggestive of Type II submucous myoma. (a) The intramural component of the myoma and, (b) the myoma component protruding into the cavity. The protrusion index is calculated by [(b/a+b) ×100].

At sonohysterography, submucosal myomas can be classified into three grades according to the following double criteria (15, 16): i. the widest diameter of fibroids in a plane perpendicular to the endometrium (sagittal planes for anterior, posterior and fundal myomas, and transverse sections for lateral myomas) and ii. the angle which is made between the myoma and the adjacent uterine wall. Sonohysterographic grading of submucosal myomas based on to these double criteria is determined as follows: grade 0=complete intracavitary fibroid, pedunculated, without intramural extension, angle ≤20˚; grade 1=sessile fibroid with ≥50% of the endocavitary part protruding into the cavity when dilated by saline, angle ≤90˚; and grade 2=endocavitary part <50%, angle >90˚ (Fig 2 A-C).

### Endometrial hyperplasia

Endometrial hyperplasia is a proliferative disorder of the endometrium that usually results from unopposed estrogenic stimulation, which causes post-menopausal bleeding in 4-8% of cases (17). Histopathologically, endometrial hyperplasia is classified as simple or complex by the presence or absence of cytological atypia. Other risk factors for developing endometrial hyperplasia are tamoxifen use, nulliparity, obesity, hypertension and diabetes (18). The risk of cancer development increases to 23% in patients with atypical hyperplasia, whereas simple hyperplasia without atypia may progress to carcinoma in 2% of cases (19).

**Fig 3:**
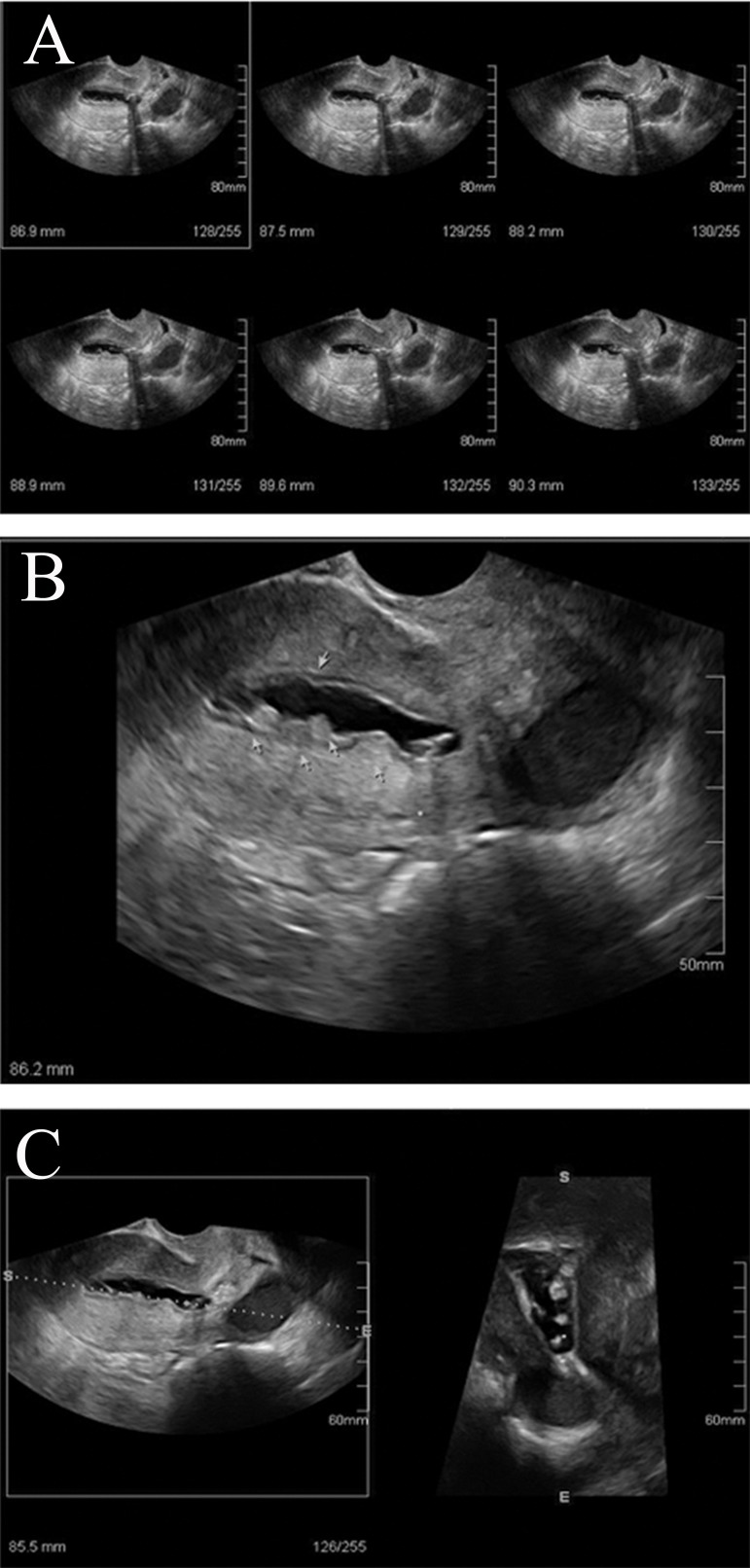
A-C. The sonohysterogram shows focal and asymmetric thickening of endometrium measuring 10 mm , presenting with smooth and spiky (cauliflower) surface. Three-dimensional rendering demonstrates better visualization. This patient had excessive bleeding. Histopathology following dilatation and curettage confirmed endometrial hyperplasia.

Endometrial hyperplasia may be suspected sonographically. The principal findings are a thickness of >15 mm or >8 mm after menopause and the presence of a non-homogenous echo pattern with microcystic changes. On sonohysterography, endometrial hyperplasia and carcinoma generally appear as an irregular, thickened, heterogeneous endometrium, which is often diffusely distributed (Fig 3A, B). Three-dimensional rendering and volumetric studies offer better visualization of focal endometrial hyperplasia, which enables the differentiation between normal proliferative and hyperplastic endometrium in patients on ART or tamoxifen therapy (20) (Fig 3A-C).

### Synechiae

**Fig 4:**
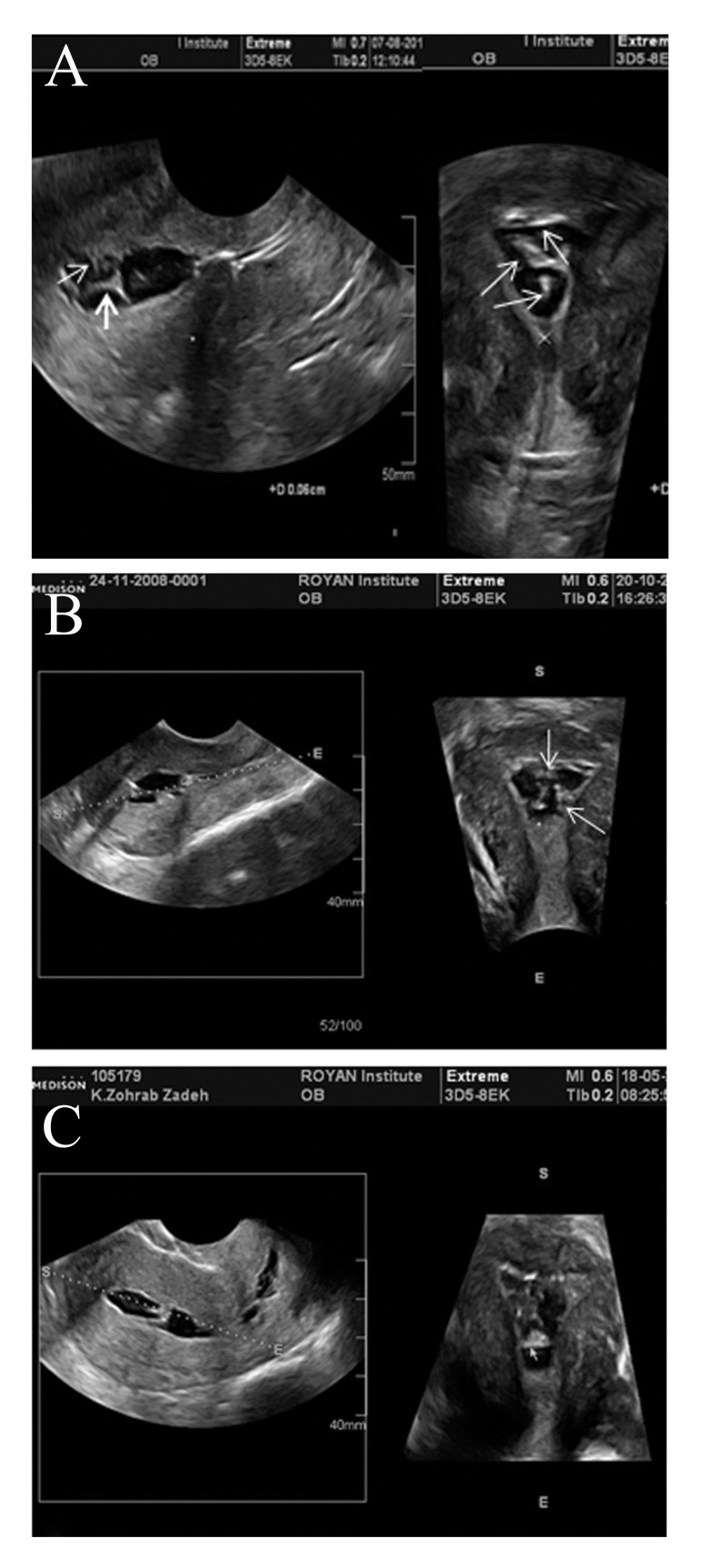
Strands of tissue cross the endometrial cavity; suggestive of endometrial synechiae. A. Sagital and coronal view of mild synechiae. Coronal section shows adhesions involve one-fourth of the uterine cavity (arrows). B. Moderate synechiae; Coronal section demonstrates adhesion involves about one-half of uterine cavity. C. Severe synechiae, Coronal section represents adhesion involves about three-fourths of uterine cavity.

Uterine synechiae or adhesions that are clinically present with infertility, recurrent abortion, and reduced menstrual flow may be minor and affect a small area of the uterine wall or extensive with diffuse involvement and obliteration of a large part of the uterine cavity. Synechiae are categorized as mild, moderate, or severe, according to whether adhesions involve one-fourth, one-half, or over three-fourths of the uterine cavity. On an ultrasound of a patient with Asherman’s syndrome, the adhesions usually appear as endometrial irregularities or hyperechoic bridges within the endometrial cavity. Three-dimensional ultrasound demonstrates a significant reduction of the endometrial cavity volume in all reformatted sections. With sonohysterography, adhesions are usually seen as echogenic (similar to the myometrium), mobile and thin or thick strands of tissue that cross the endometrial cavity, attaching to both uterine walls (10) (Fig 4 A-C). In the presence of synechiae, the uterine cavity is often not completely filled during sonohysterography.

## Conclusion

Although hysteroscopy is the gold standard in the detection of intrauterine pathologies, particularly in patients with infertility, three-dimensional MS sonohysterography (3D-MS-SHG) offers a good overall agreement with diagnostic hysteroscopy over conventional three-dimensional multi-planar views. Uterine volume sampling, simultaneous analysis of three orthogonal planes and rendering of images allow better estimation of shape, size, location and protruding degree of endometrial lesions. 3D-MS-SHG, as a less invasive and more cost effective alternative to diagnostic hysteroscopy, is a reliable, accurate method that should be considered for precise pre-operative assessment.
